# WeldVGG: A VGG-Inspired Deep Learning Model for Weld Defect Classification from Radiographic Images with Visual Interpretability

**DOI:** 10.3390/s25196183

**Published:** 2025-10-06

**Authors:** Gabriel López, Pablo Duque Ramírez, Emanuel Vega, Felix Pizarro, Joaquin Toro, Carlos Parra

**Affiliations:** 1 Escuela de Ingeniería Informática, Pontificia Universidad Católica de Valparaíso (PUCV), Av. Brasil 2241, Valparaíso 2362807, Chile; gabriel.lopez.m@mail.pucv.cl (G.L.); emanuel.vega@pucv.cl (E.V.); joaquin.toro.g@mail.pucv.cl (J.T.); 2 Departamento de Mecánica, Universidad Técnica Federico Santa María, Av. Federico Sta. María 6090, Viña del Mar 2520000, Chile; felix.pizarro@usm.cl (F.P.); carlos.parram@usm.cl (C.P.)

**Keywords:** weld inspection, radiographic inspection, convolutional neural network, visual testing, industrial AI, Grad-CAM++, interpretability, weld defect classification

## Abstract

Visual inspection remains a cornerstone of quality control in welded structures, yet manual evaluations are inherently constrained by subjectivity, inconsistency, and limited scalability. This study presents WeldVGG, a deep learning-based visual inspection model designed to automate weld defect classification using radiographic imagery. The proposed model is trained on the RIAWELC dataset, a publicly available collection of X-ray weld images acquired in real manufacturing environments and annotated across four defect conditions: cracking, porosity, lack of penetration, and no defect. RIAWELC offers high-resolution imagery and standardized class labels, making it a valuable benchmark for defect classification under realistic conditions. To improve trust and explainability, Grad-CAM++ is employed to generate class-discriminative saliency maps, enabling visual validation of predictions. The model is rigorously evaluated through stratified cross-validation and benchmarked against traditional machine learning baselines, including SVC, Random Forest, and a state-of-the-art architecture, MobileNetV3. The proposed model achieves high classification accuracy and interpretability, offering a practical and scalable solution for intelligent weld inspection. Furthermore, to prove the model’s ability to generalize, a test on the GDXray was performed, yielding positive results. Additionally, a Wilcoxon signed-rank test was conducted separately to assess statistical significance between model performances.

## 1. Introduction

Ensuring weld quality is a fundamental requirement in industrial manufacturing, particularly in structural and pressure-retaining applications where joint integrity directly impacts safety and reliability [1]. Traditionally, visual inspection serves as the first, and often the only, non-destructive testing method applied in many production environments [2]. However, despite its widespread use, manual visual inspection is inherently constrained by human variability, fatigue, and inconsistency, which can result in undetected defects, increased operational risks, and costly rework [3].

Recent advances in artificial intelligence (AI) and computer vision have opened new possibilities for augmenting or replacing manual inspection processes with automated systems that offer greater consistency, objectivity, and scalability. Among these, Convolutional Neural Networks (CNNs) have demonstrated remarkable performance in image classification tasks, including weld bead analysis and defect detection [4,5]. These models can extract hierarchical visual features directly from raw image data, making them highly effective for identifying surface-level discontinuities such as porosity, cracks, spatter, and undercuts. Despite these technological advancements, successful deployment of AI-based inspection systems in industrial settings requires more than classification accuracy alone. Interpretability becomes a critical factor to support decision-making and to build trust among operators, engineers, and quality control personnel. In response to this challenge, the present work introduces a visual inspection framework based on CNNs for automatic classification of weld defects in radiographic images. The system integrates Grad-CAM++ [6] to produce class-discriminative visual explanations for each prediction, improving transparency and enabling post hoc validation. Additionally, the model is benchmarked against both lightweight CNN variants and classical machine learning baselines such as SVM and Random Forest. To assess the robustness of the approach, statistical significance testing is performed using the Wilcoxon signed-rank test across multiple cross-validation folds.

The contributions of this work are as follows:

- A custom VGG-inspired CNN for radiographic weld defect classification, trained from scratch on the publicly available RIAWELC dataset, achieving near-perfect performance across three defect classes plus one no-defect class.
- A comparative benchmark of the CNN against lightweight deep architectures and classical machine learning baselines, evaluated under a standardized experimental protocol with stratified 10-fold cross-validation and a held-out test set.
- The integration of Grad-CAM++ visual explanations and the ROAD interpretability metric, providing both quantitative and qualitative insights into the model’s decision-making process.
- Statistical validation via Wilcoxon signed-rank tests, confirming the significance of observed performance differences and enhancing the reliability of the findings for industrial adoption.

The remainder of this paper is structured as follows: Section 2 reviews related work in AI-based weld inspection; Section 3 details the proposed CNN architecture and interpretability strategy; Section 4 presents the dataset, training protocol, and results; Section 5 concludes with a discussion on implications and future research directions.

## 2. Related Work

### 2.1. Classical Machine Learning Approaches to Image Classification

Artificial intelligence has gained increasing attention in the field of weld inspection, particularly for automating the detection of visual discontinuities. Early approaches relied on classical machine learning models such as Support Vector Machines (SVMs) and Random Forests (RFs), which classified weld defects based on handcrafted features such as texture, intensity, or geometric descriptors [7,8]. For instance, Mu et al. [9] applied Principal Component Analysis (PCA) to reduce feature dimensionality before classifying weld imperfections using SVM, achieving promising results on ultrasonic inspection signals. However, these methods typically required manual feature engineering and were sensitive to external variations, such as illumination or surface irregularities, limiting their robustness in industrial contexts.

### 2.2. Convolutional Neural Networks Approaches to Defects Classification

In this regard, more recent approaches have incorporated computer vision strategies led by Convolutional Neural Networks (CNNs), which demonstrate superior performance in weld defect classification due to their ability to learn hierarchical spatial features directly from image data. A recent systematic literature review by Cumbajin et al. [10] surveyed 59 empirical studies on CNN-based surface defect detection—including metallic surfaces—and highlighted that over 80% employ transfer learning, more than 60% use data augmentation, and many still struggle with real-world variability in lighting and surface conditions. This body of work confirms that CNNs are now the dominant paradigm in modern defect detection research, although challenges related to data availability and interpretability remain unresolved. In the specific context of weld inspection, these challenges are particularly critical, as surface conditions can vary widely due to material composition, welding technique, and environmental exposure, even under industrial regulations. For instance, in [11], the authors demonstrated that fluctuations in wind speed, humidity, and temperature during field welding significantly influence defect formation and quality, underscoring the importance of models robust to environmental variability. Furthermore, regulatory compliance and traceability are essential in industrial quality control, demanding systems that are not only accurate but also interpretable and aligned with established standards such as AWS D1.1 [1] and ISO 13018 [2]. In [12], the authors presented WeldNet, a lightweight CNN model tailored for welding defect recognition. Their network uses ensemble distillation to improve generalization and achieves an 8.9% higher accuracy compared to ResNet-18 while reducing the number of parameters to only 26.8% and reaching 41 FPS on a CPU, demonstrating high inference speed. Similarly, Biasuz Block et al. [13] introduced LoHi-WELD, one of the few publicly available industrial datasets for weld defect detection, comprising 3022 annotated images captured using MAG welding robots under real operating conditions. The authors employed a YOLOv7-tiny-based model, achieving 0.77 mean Average Precision (mAP) in coarse classification and 0.69 mAP in fine-grained defect recognition while maintaining real-time inference capabilities on edge devices. Also, Hou et al. [14] presented a transfer-learning approach using a pre-trained MobileNet model (on ImageNet), which they fine-tuned for welding defect classification on a custom dataset of 5000 images across four defect types. To address class imbalance and accelerate convergence, they integrated DropBlock regularization and Global Average Pooling (GAP). Their model achieved a high accuracy of 97.7%, outperforming baseline CNNs like Xception, VGG-16, and ResNet-50. In general, despite these advances, many existing approaches remain limited in scope. Most rely on laboratory-acquired or radiographic datasets with constrained variability and do not consider industrial quality standards like AWS D1.1 or ISO 13018.

### 2.3. Welding Images Classification Through Segmentation Techniques

An important branch of image classification relies on segmentation techniques to delineate weld regions and group image pixels for defect identification. Recent advances have integrated deep learning into these approaches, greatly improving their robustness and accuracy. For example, Chang and Wang [15] proposed a semantic segmentation-based method that combines cylindrical projection with an improved SegNet architecture, enabling better recognition of small-size defects in radiographic images by increasing the proportion of defect pixels and mitigating background noise. Similarly, Xu et al. [16] introduced an end-to-end defect detection framework based on an FPN-ResNet-34 semantic segmentation network, which not only improved pixel-level boundary extraction but also incorporated optimization strategies such as data augmentation and hybrid loss functions to mitigate class imbalance, achieving accurate discrimination of defect types and boundaries in radiographic images. Such segmentation-based strategies not only enhance pixel-level defect localization but also offer stronger interpretability for industrial inspection, though they often come at the cost of higher computational complexity compared to standard classification pipelines. These computational trade-offs become nuanced when leading concerns focus on reducing the computational cost [17].

### 2.4. Multimodal Image Classification in Welding

Recent research has highlighted the potential of multimodal learning to advance welding defect detection by integrating heterogeneous data sources such as images, current, voltage, and process parameters. For instance, Jiang et al. [18] proposed a ResNet-Transformer model for aluminum alloy TIG welding that fuses molten pool images with welding current and speed, achieving nearly 99% accuracy through multimodal factorized bilinear fusion and image enhancement strategies. In the same manner, Mustafaev (2024) [19] worked in an advanced multimodal approach for Gas Metal Arc Welding Quality Control. This work consisted on combining high quality images of weld defects with sensor readings of voltage, current, and gas flow rate to train deep learning models to detect weld defects. The employed pretrained EfficientNetB2 and a ResNet50 yielded high results, 97.33% and 98.67% accuracy, respectively. Another novel approach to multimodal weld defect detection can be found in Stavropoulos’s (2022) [20] work assessing the quality on Submerged Arc Welding (SAW) by utilizing multimodal monitoring. Using both IR and RGB cameras, the work proposed to classify defects on SAW with a deep learning CNN model. This proposed 2-branch CNN managed to produce very positive results, reaching 99.7%, 99.1%, 100%, and 97.9% accuracy on each of the four classes. Such approaches demonstrate that combining visual and temporal signals yields more robust feature representations, capturing subtle variations that uni modal systems often miss. Nonetheless, the reliance on synchronized high-quality sensor data and the elevated computational demands of fusion architectures present practical obstacles to industrial adoption. Thus, while multimodal methods clearly improve classification accuracy in controlled experiments, their real-world deployment remains constrained by data availability, cost, and scalability considerations.

### 2.5. Explainable Artificial Intelligence in CNN Image Classification

Furthermore, interpretability is rarely addressed, even though decision traceability is essential in safety-critical contexts. Techniques like Grad-CAM++ [6] provide valuable insights into model decisions but are seldom integrated into real-world weld inspection pipelines. Thus, despite algorithmic advances, a significant gap persists between academic prototypes and deployable industrial solutions. Industry surveys indicate that most models rely on limited or synthetic datasets, often insufficient to handle environmental variability, and deployment lag can reach three years from publication to real-world use [21]. Moreover, deep-learning applications in fault diagnosis emphasize the critical need for explainability, as transparency is essential to foster trust and enable effective intervention in maintenance workflows [22]. Consequently, there is growing demand for lightweight, explainable, and standards-compliant models capable of operating reliably under realistic conditions and supporting predictive maintenance strategies.

## 3. Proposed Approach

This section describes the methodology developed to perform automatic classification of weld defects from visual data. The proposed approach combines a custom-designed Convolutional Neural Network (CNN), tailored for efficiency and interpretability, with a post hoc visual explanation mechanism based on Grad-CAM++ (pytorch-grad-cam v1.5.5). The overall design aims to satisfy industrial inspection requirements and facilitate transparent decision-making.

### 3.1. Convolutional Neural Networks: Principles and Components

Convolutional Neural Networks (CNNs) are deep learning architectures specifically designed to process grid-like data, such as images, by automatically learning hierarchical spatial representations [23,24]. The fundamental operation in a CNN is the convolution, where learnable filters (or kernels) are applied across the input image to extract local patterns like edges, textures, or shapes. Mathematically, this operation can be expressed as follows [3]:

$$Y\left(i,j\right)=\sum_{m=0}^{M-1}\sum_{n=0}^{N-1}K\left(m,n\right)\cdot X\left(i+m,j+n\right),$$

where *X* represents the input image, *K* is the convolutional kernel, and *Y* is the resulting feature map. To introduce non-linearity, each convolutional output is passed through an activation function, typically the Rectified Linear Unit (ReLU), defined as ReLU(x)=max(0,x).

Subsequently, pooling layers reduce the spatial dimensionality of the feature maps, enhancing translation invariance and reducing computational load. Max pooling, in particular, retains the most significant local activation via a simple maximum operation:

$$Y=\max_{(i,j)\in R}X\left(i,j\right).$$

The resulting compact feature representations are flattened and passed through one or more fully connected layers, which integrate the learned features to perform high-level classification, culminating in a softmax layer that outputs class probabilities. CNNs are trained via backpropagation and optimized using algorithms, such as stochastic gradient descent or Adam, with the aim of minimizing a loss function (e.g., cross-entropy). Crucially, CNNs leverage weight sharing and local receptive fields, significantly reducing the number of trainable parameters and improving generalization. This architectural design allows CNNs to learn low-level features in early layers (e.g., edges and corners) and increasingly abstract representations in deeper layers, making them particularly suitable for industrial visual inspection tasks such as weld defect classification, where both fine-grained local cues and global structural information are essential.

### 3.2. Convolutional Neural Networks: Architectural Advantages

Although recent architectures such as Transformers and hybrid networks have gained attention, Convolutional Neural Networks (CNNs) remain particularly advantageous for the proposed application. Firstly, CNNs are computationally efficient due to weight sharing and local connectivity, which allow them to achieve high accuracy on medium-sized datasets without requiring the scale of data or training resources demanded by Transformers. This efficiency has been consistently highlighted in domains with limited data availability, such as computer vision, where CNNs remain the state-of-the-art for image detection and segmentation tasks [25,26].

In addition to efficiency, CNNs offer a higher degree of interpretability. Their spatially structured feature maps enable visual explanation methods (e.g., Grad-CAM), which are more transparent and easier to align with human expertise than the distributed attention patterns of Transformer models. This property has been emphasized in both medical diagnostics [25] and agricultural inspection [27], where accountability and operator trust are critical.

Finally, CNNs are well-matched to the localized and texture-based patterns present in radiographic weld images. Similar to how they capture subtle anomalies in X-rays [26] or diseased crop leaves [27], CNNs can hierarchically extract edges, pores, and cracks in welds, which are inherently local defects. More recent architectures, while powerful, are not optimized for such localized feature hierarchies and impose greater computational and data burdens [26]. For these reasons, CNNs provide the most suitable and theoretically grounded choice for our proposed defect detection framework.

### 3.3. Model Interpretability via Grad-CAM++

To improve model transparency and support human-in-the-loop decision-making, this work integrates Grad-CAM++ as a visual explanation mechanism. Grad-CAM++ is an extension of the original Gradient-weighted Class Activation Mapping (Grad-CAM), designed to generate class-discriminative saliency maps from any convolutional layer of a CNN [6,28].

The technique works by computing the weighted average of the feature maps in a selected convolutional layer, where the weights are derived from the gradients of the target class score with respect to each activation. Unlike its predecessor, Grad-CAM++ is better suited for scenarios involving multiple object instances or fine-grained visual differences, as it accounts for higher-order gradients and produces sharper and more localized visualizations.

Formally, for a given class *c*, the saliency map $L_{\mathrm{Grad}-\mathrm{CAM}++}^{c}$ is computed as follows:

$$L_{\mathrm{Grad}-\mathrm{CAM}++}^{c}=\mathrm{ReLU}\left(\sum_{k}\alpha_{k}^{c}A^{k}\right),$$

where $A^{k}$ is the *k*-th activation map of the selected convolutional layer, and $\alpha_{k}^{c}$ are the weights derived from the gradients $\frac{\partial^{2}y^{c}}{\partial {\left(A_{ij}^{k}\right)}^{2}}$ and higher-order terms. By overlaying the resulting heatmaps on the original input images, Grad-CAM++ helps identify which regions of the weld image influenced the model’s prediction, thereby increasing interpretability and offering valuable insights for expert validation. This is particularly relevant in regulated inspection environments, where explainability is essential for accountability and user trust.

### 3.4. CNN-Based Weld Defect Classifier

**Figure 1:**
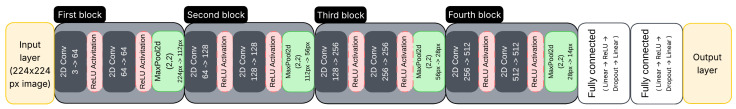
Proposed WeldVGG layer diagram.

A deep Convolutional Neural Network (CNN) architecture, inspired by the VGG family of models, was proposed for the task of weld defect classification. This model, referred to as WeldVGG, was implemented from scratch, without the use of pretrained weights or transfer learning, and trained using a standardized experimental protocol. As illustrated in Figure 1, WeldVGG consists of four convolutional blocks, each containing two convolutional layers with 3 × 3 kernels and ReLU activations, followed by a 2 × 2 max pooling layer for spatial downsampling. The number of filters increases progressively across blocks (64, 128, 256, 512), enabling hierarchical extraction of complex spatial and textural features from weld images. Padding was used to preserve spatial dimensions throughout the convolutional layers. Following the convolutional backbone, the output is flattened and passed through three fully connected layers. The first two dense layers contain 512 and 256 units, respectively, each followed by ReLU activation and dropout (*p* = 0.5) for regularization. The final classification layer outputs probabilities over the four defect categories: cracking (CR), porosity (PO), lack of penetration (LP), and no defect (ND). This architecture contains approximately 56 million trainable parameters and is designed to offer high representational capacity for detailed defect recognition. A complete layer-by-layer summary is presented in Table 1.

In addition to WeldVGG, a modern lightweight CNN, MobileNetV3 [29], was employed as a parameter-efficient comparator. MobileNetV3 is designed for high accuracy under strict computational constraints, making it well-suited for deployment in resource-limited industrial environments. It combines depthwise separable convolutions with inverted residual blocks, squeeze-and-excitation attention mechanisms, and the hard-swish activation function to achieve strong representational capacity at a fraction of the parameter count of traditional CNNs. Unlike WeldVGG, which prioritizes representational depth, MobileNetV3 emphasizes computational efficiency, containing only 1.5 million parameters in its small variant and 5.4 million in its large variant, depending on configuration. This complementary architecture was included to assess whether lightweight, state-of-the-art CNNs can match the accuracy of heavier custom designs in weld defect classification.

### 3.5. Input Preprocessing

**Figure 2:**
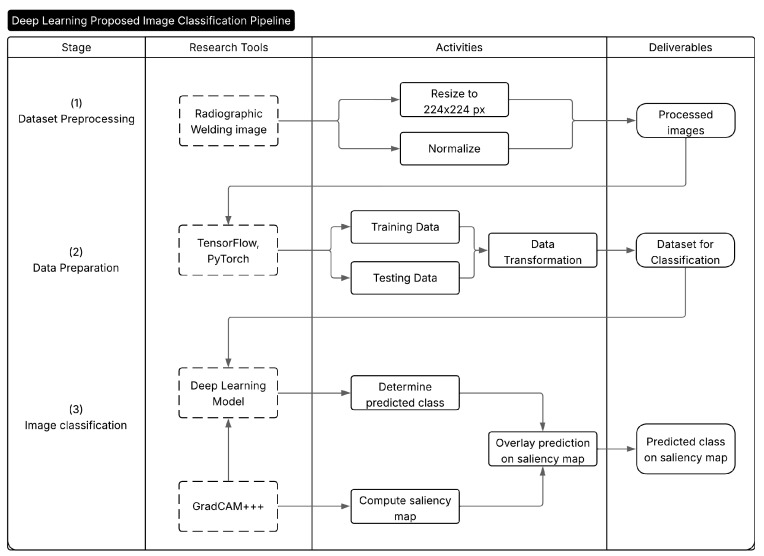
Deep learning proposed image classification pipeline.

For the CNN-based models, all input images were first resized to a resolution of 224×224 pixels. The images were then converted to PyTorch (version 2.6.0+cu124) tensors and normalized channel-wise using a fixed mean and standard deviation of 0.5 for each RGB channel. This transformation scaled pixel values from the original [0,255] range to a standardized [−1,1] interval, which is suitable for stable convergence during CNN training. The resulting standardized tensors were then passed through the convolutional network described in Section 3.3. Algorithm 1 outlines the complete inference and visualization pipeline, from image input to Grad-CAM++ explanation. Figure 2 illustrates the pipeline in a visual manner, each Stage requires the Research Tools, which are transformed by the Activities and turned into Deliverables.
**Algorithm 1** Proposed Weld Defect Classification and Visualization Pipeline**Require:** Input image *I***Ensure:** Predicted class label $\hat{y}$, corresponding Grad-CAM++ saliency map $M_{\hat{y}}$  1:**Initialization:** Set preprocessing parameters (resize to 224×224, normalize to [−1,1])  2:Preprocess input image *I* using the defined parameters  3:Feed *I* into CNN and compute class probabilities p=CNN(I)  4:Determine predicted class $\hat{y}=\arg\max\left(p\right)$  5:Compute Grad-CAM++ saliency map $M_{\hat{y}}$ for predicted class $\hat{y}$  6:Overlay $M_{\hat{y}}$ on *I* to generate the visual explanation $\tilde{I}$  7:**Termination:** Stop when $\hat{y}$ and $M_{\hat{y}}$ are obtained  8:**return** $\hat{y}$, $M_{\hat{y}}$, $\tilde{I}$

## 4. Experimental Results

### 4.1. RIAWELC Dataset

The experiments in this study were conducted using a labeled dataset of weld radiographic images, compiled and annotated in prior works [30,31]. The images in this dataset were compiled using industry-standard X-Ray equipment in a real non-disclosed manufacturing environment. While the original publication does not include the specific equipment and its parameters, its images reflect real industrial inspection scenarios aligned with these standards, thereby supporting its suitability as a benchmark dataset for image classification.

The dataset comprises 24,407 grayscale radiographic images, each classified into one of four categories: three defect types, cracking (CR), porosity (PO), lack of penetration (LP), and a no defect (ND) category. These classes represent common issues encountered in weld inspections and are critical for ensuring structural integrity in industrial manufacturing. Each sample in the dataset corresponds to a cropped region of interest, standardized to a size of 224 × 224 pixels, extracted from larger weld radiographs. The annotations were performed by domain experts and reflect real-world variations in defect shape, contrast, and noise, making the dataset a representative benchmark for defect classification. Furthermore, these classes are consistent with the categories of discontinuities defined in major international standards for weld inspection. For example, the AWS D1.1 [1] Structural Welding Code explicitly lists cracks, porosity, and incomplete penetration as rejectable discontinuities due to their impact on weld integrity. Similarly, ISO 17636-1 [32] provides guidelines for radiographic testing techniques, while ISO 10675-1 [33] defines acceptance levels for such discontinuities. This alignment reinforces the dataset’s relevance for industrial applications, as summarized in Table 2.

**Figure 3:**
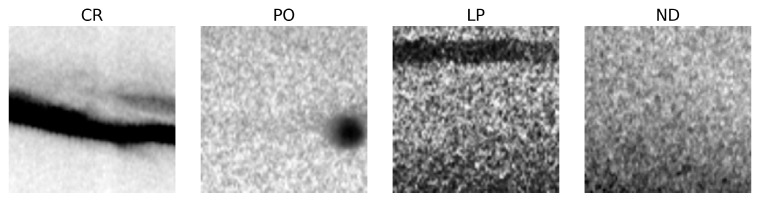
Representative examples of weld radiographic images from each defect class: Cracking (CR), Porosity (PO), Lack of Penetration (LP), and No Defect (ND).

The dataset was initially stratified by class into training (65%), validation (25%), and testing (10%) subsets. Table 3 summarizes the class distribution across these splits. A representative example from each class is shown in Figure 3.

The proposed WeldVGG is specifically suited to this dataset due to CNNs being particularly well suited to this dataset because the grayscale radiographs encode weld defects as localized edge- and texture-based patterns (e.g., crack tips, pore clusters, root gaps) that CNNs learn effectively through small receptive fields and hierarchical feature stacking. Crucially, the dataset’s scale (24,407 annotated tiles across CR/PO/LP/ND) provides sufficient supervised signal for training compact or moderate-capacity CNNs, and prior experiments on the same corpus [30,31] have already demonstrated high test accuracy with a lightweight model, indicating that defect classes are separable in a CNN feature space. These properties make CNNs a natural and empirically validated choice for this benchmark.

### 4.2. GDXray Weld Subset (Series W0003)

To evaluate cross-dataset generalization, we employed the weld subset of the GDXray database [34]. While the welding category of GDXray includes four distinct series (W0001–W0004), only Series W0003 was used in this study, as it provides digitized radiographs from a round-robin test conducted by BAM under ISO 17636-1 standards, together with the accompanying annotation file.

Series W0003 contains 68 radiographs in total, of which 38 images include detailed defect annotations in the Excel metadata. Each annotated radiograph was manually partitioned into 1 cm-wide segments (248 pixels at the native resolution) which were later resized to 224 × 224 pixels following the same convention as in the RIAWELC dataset. From these crops, labels were manually assigned based on the defect type reported in the annotation file.

**Figure 4:**
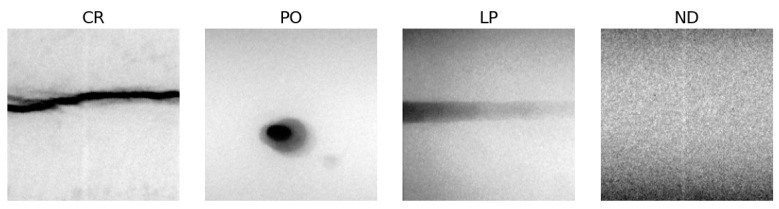
Representative cropped samples from the curated GDXray W0003 subset, resized to 224×224 pixels. The four classes considered were Cracking (CR), Porosity (PO), Lack of Penetration (LP), and No Defect (ND).

For consistency with the RIAWELC setup, only the four common defect categories were considered: Cracking (CR), Porosity (PO), Lack of Penetration (LP), and No Defect (ND). Crops containing multiple simultaneous defect types were discarded to avoid label ambiguity. After this filtering step, a balanced subset of 120 images (30 per class) was constructed for evaluation, the distribution of defect types is shown in Table 4. This curated subset ensured compatibility with the RIAWELC experimental setup, yet preserved the intrinsic variability of GDXray radiographs, providing a more realistic test of cross-dataset generalization. A sample of these labeled images can be seen in Figure 4.

### 4.3. Experimental Setup

**Figure 5:**
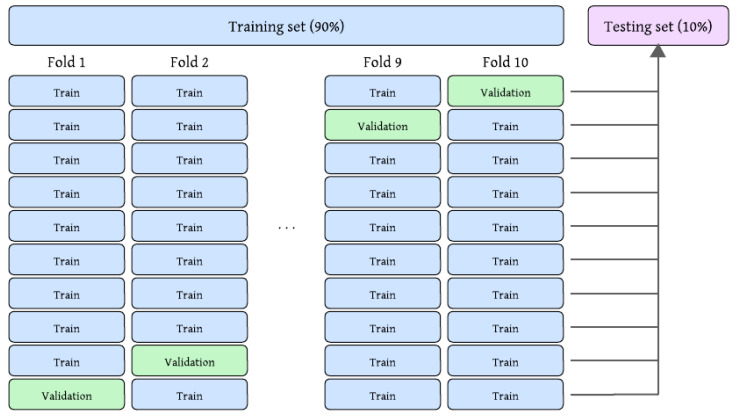
Training/testing setup used in the experimentation.

All experiments were conducted using the dataset described in Section 4.1. The original training and validation splits were merged into a combined set of 21,964 images, preserving class balance. A 10-fold stratified cross-validation protocol was applied to this set for model training and validation, ensuring that each image was used once for validation and nine times for training. A fixed holdout test set comprising 2443 samples (10% of the total data) was reserved for final evaluation, as depicted in Figure 5.

The primary architecture investigated is WeldVGG, a VGG-inspired Convolutional Neural Network (CNN) implemented from scratch in PyTorch (see Section 3.4). To provide a modern and computationally efficient baseline, MobileNetV3 [29] was also included for comparison. MobileNetV3 leverages depthwise separable convolutions, inverted residual blocks, and squeeze-and-excitation attention to achieve high accuracy with substantially fewer parameters than WeldVGG, making it suitable for deployment in resource-constrained environments. For both CNNs, input images were resized to 224×224 pixels and normalized prior to training.

Additionally, four traditional classifiers—Decision Tree, Random Forest, K-Nearest Neighbors (KNN), and Support Vector Classifier (SVC)—were implemented using Scikit-learn. For these models, images were resized to a smaller 150×150 resolution to reduce computational cost and feature dimensionality. The resized images were flattened into one-dimensional vectors, standardized, and compressed via PCA to 100 components prior to training. Hyperparameter tuning was performed via GridSearchCV with 5-fold cross-validation on the training folds. The explored parameter ranges are provided in Table A1, and the optimal configurations found for each classifier are summarized in Table A2 (Appendix A).

Both CNN architectures were trained for 30 epochs per fold using the Adam optimizer ($1\times 10^{-4}$ learning rate), cross-entropy loss, and a batch size of 32. Model weights were saved based on the highest validation F1-score. Traditional classifiers were similarly selected based on their best F1-score during tuning. A concise overview of all experimental settings is provided in Table 5.

All experiments were conducted using Kaggle’s hosted environment. For training the CNN architectures, the runtime was configured with a NVIDIA T4 GPU (×2), 16 virtual CPUs, and 30 GB of RAM. Traditional machine learning models were executed on CPU resources.

### 4.4. Evaluation Metrics

The classification performance of the proposed and baseline models was evaluated using standard metrics, including accuracy, precision (*P*), recall (*R*), and F1-score (*F*1), the harmonic mean of precision and recall. These metrics are computed from the confusion matrix, based on the number of correctly and incorrectly predicted class labels across all test samples. Precision is defined as the proportion of correctly predicted positive samples among all samples predicted as positive for a given class, while recall quantifies the proportion of actual positive samples that were correctly identified. The F1-score, which balances both measures, is computed as follows:

$$F1=\frac{2}{\frac{1}{P}+\frac{1}{R}}=2\cdot \frac{P\cdot R}{P+R}.$$

Additionally, overall accuracy is reported, defined as the ratio of total correct predictions to the total number of samples:

$$\mathrm{Accuracy}=\frac{TP+TN}{TP+TN+FP+FN},$$

where TP, TN, FP, and FN refer to true positives, true negatives, false positives, and false negatives, respectively.

For multi-class classification, all metrics were computed using macro-averaging, which calculates the unweighted mean of each metric across all classes. This approach treats all classes equally, regardless of their frequency, and is particularly appropriate in the presence of class imbalance. For robust test evaluation, we trained 10 models using stratified 10-fold cross-validation on the training set. Each model was selected based on its best validation F1-score within its fold. All 10 trained models were then independently evaluated on the same holdout test set (10% of data). Final metrics are reported as mean ± standard deviation over these 10 evaluations.

To quantitatively evaluate the interpretability of the Grad-CAM++ visualizations, we adopted the ROAD (Remove And Debias) metric [35], a class-sensitive approach designed to measure the faithfulness of saliency maps. The ROAD metric assesses the impact of iteratively removing the most or least relevant pixels, as indicated by the saliency map, and recording the resulting change in the model’s confidence for the predicted class. Specifically, we employ the ROADCombined variant, which aggregates performance degradation across multiple removal thresholds (e.g., 20%, 40%, 60%, 80%), yielding a single scalar score per sample. This variant was implemented using the pytorch-grad-cam library [36]. The ROAD score is defined as follows:

(1) $$\mathrm{ROAD}\;\mathrm{Score}=\frac{\mathrm{Least}\;\mathrm{Relevant}\;\mathrm{First}-\mathrm{Most}\;\mathrm{Relevant}\;\mathrm{First}}{2},$$

where higher positive values indicate that the most relevant pixels, as identified by the saliency map, have a strong influence on the model’s confidence, thereby validating the quality of the visual explanation. Conversely, near-zero or negative values suggest weak localization or misleading attribution.

### 4.5. Results

**Figure 6:**
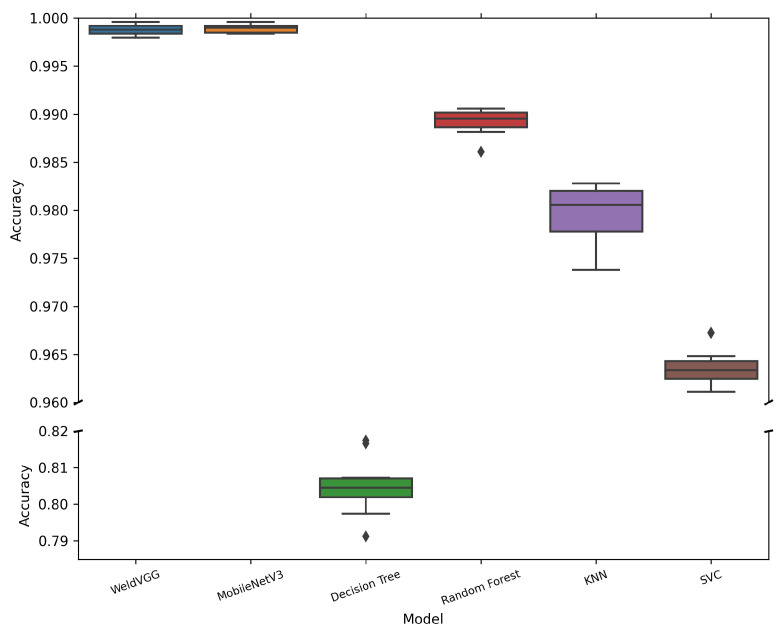
Distribution of macro-averaged Accuracy scores obtained by the 10 models trained in each cross-validation fold and subsequently evaluated on the independent holdout test set. A broken y-axis is applied to improve the visibility of narrow interquartile ranges for high-performing models.

**Figure 7:**
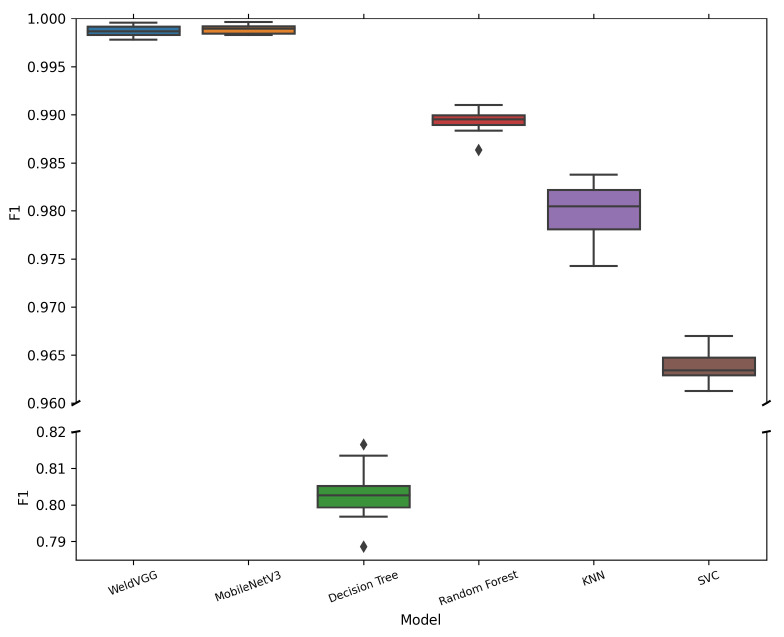
Distribution of macro-averaged F1-scores obtained by the 10 models trained in each cross-validation fold and subsequently evaluated on the independent holdout test set. A broken y-axis is applied to improve the visibility of narrow interquartile ranges for high-performing models.

**Figure 8:**
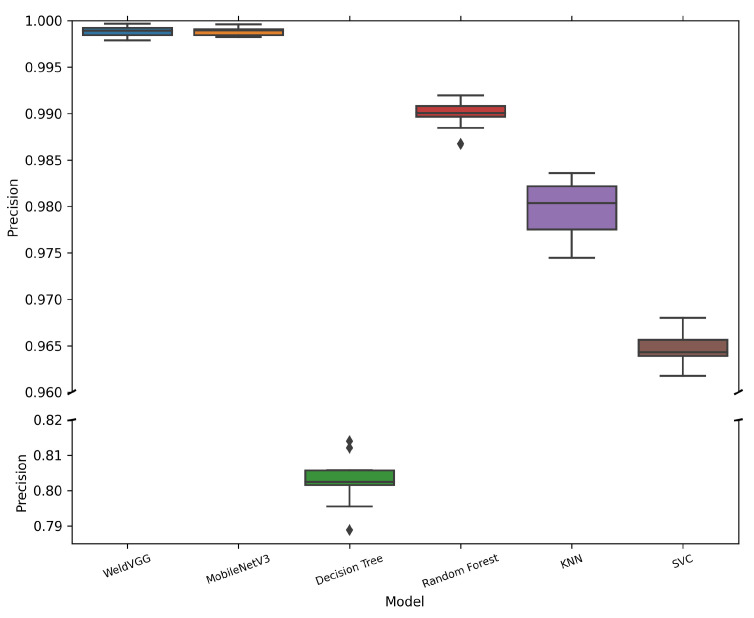
Distribution of macro-averaged Precision scores obtained by the 10 models trained in each cross-validation fold and subsequently evaluated on the independent holdout test set. A broken y-axis is applied to improve the visibility of narrow interquartile ranges for high-performing models.

**Figure 9:**
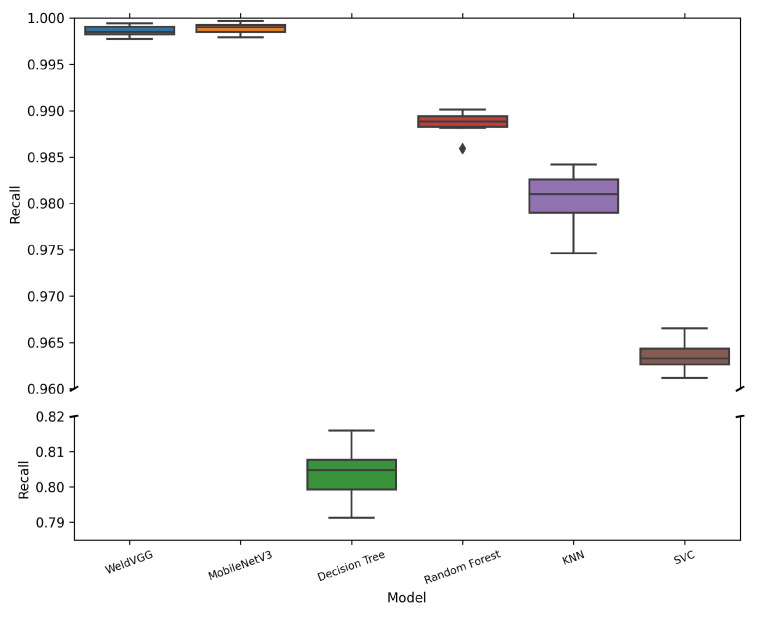
Distribution of macro-averaged Recall scores obtained by the 10 models trained in each cross-validation fold and subsequently evaluated on the independent holdout test set. A broken y-axis is applied to improve the visibility of narrow interquartile ranges for high-performing models.

Table 6 summarizes the mean and standard deviation of macro-averaged metrics across 10 cross-validation folds for all evaluated models. Both deep learning models, MobileNetV3 and WeldVGG, achieved near-perfect classification results, substantially outperforming traditional machine learning baselines. MobileNetV3 slightly surpassed WeldVGG across all metrics, confirming the effectiveness of modern lightweight architectures when applied to weld radiographs. Nevertheless, WeldVGG remained highly competitive, with marginal differences that were within statistical variation. In contrast, the shallow baselines exhibited a clear performance gap, with Random Forest providing the strongest results among them, followed by KNN, SVC, and Decision Tree. The boxplots in Figure 6, Figure 7, Figure 8 and Figure 9 provide further insight into the stability and variability of these outcomes across folds.

Figure 6 illustrates the distribution of macro-averaged Accuracy values across the evaluated models. Both WeldVGG and MobileNetV3 achieved near-perfect performance across all folds, with median accuracies of 0.999 and extremely narrow interquartile ranges (IQR < 0.002). The absence of significant outliers indicates highly stable predictions, underscoring the ability of deep CNN architectures to consistently extract robust spatial features from weld radiographs.

Among the shallow baselines, Random Forest delivered the strongest results, though with slightly greater dispersion across folds, likely due to the fixed, low-dimensional feature space generated through PCA compression. KNN exhibited lower accuracy and wider variability, reflecting its sensitivity to data distribution shifts across folds. The SVC model showed even lower accuracy, with several folds producing outliers that suggest instability in decision boundaries. Finally, Decision Tree attained the lowest accuracy overall, with broad dispersion and multiple outliers, confirming its limited suitability for such complex visual classification tasks.

Figure 7 presents the distribution of macro-averaged F1-scores across all models, offering a deeper view into class-level consistency by balancing both precision and recall. Both WeldVGG and MobileNetV3 achieved near-perfect and highly stable F1-scores across all folds, confirming that their strong accuracy was not simply driven by majority class predictions but reflected consistent performance across all defect categories. The narrow interquartile ranges and absence of significant outliers demonstrate that both deep CNNs generalize reliably across folds.

Among the traditional baselines, Random Forest maintained the highest F1 performance, though with slightly wider dispersion and occasional outliers, suggesting occasional difficulties with minority class samples. KNN followed with competitive but more variable results, reflecting sensitivity to fold composition and intra-class variation. The SVC model exhibited lower overall F1-scores with a tighter distribution, indicating consistent but limited capacity to capture defect-specific patterns. Finally, Decision Tree showed the lowest and most unstable F1 performance, underscoring its inadequacy for complex weld radiograph classification.

Figure 8 shows that MobileNetV3 and WeldVGG consistently achieved the highest precision, with median values near 0.999 and extremely narrow interquartile ranges. This indicates that both CNNs are highly effective at minimizing false positives across defect categories. MobileNetV3 displayed slightly lower variability compared to WeldVGG, suggesting even more consistent decision boundaries. Such reliability is particularly important in industrial inspection, where false alarms can trigger unnecessary weld rework and production delays. Random Forest, while generally precise, exhibited occasional drops in precision, possibly linked to the inherent information loss caused by PCA compression or the model’s sensitivity to fold-specific distributions.

Finally, as shown in Figure 9, both WeldVGG and MobileNetV3 achieved near-perfect recall with negligible variability, reliably capturing true defect instances across all classes and folds. This high sensitivity is critical in weld inspection, as missed defects (false negatives) directly compromise structural integrity. The narrow interquartile ranges and absence of outliers highlight the robustness of both CNNs in consistently identifying defective regions. Among the baselines, Random Forest attained the strongest recall, though with occasional drops that suggest susceptibility to class imbalance and feature compression. KNN also performed reasonably well but exhibited broader variability, indicating dependence on data distribution across folds. SVC displayed lower overall recall, reflecting its tendency to miss defect cases. Decision Tree again showed the weakest recall, with both low scores and high variability, underscoring its inability to generalize for reliable defect detection.

Overall, the boxplot analysis shows that the depth and representational power of CNN architectures, particularly WeldVGG and MobileNetV3, translate into both higher average scores and greater stability across folds. This consistency is crucial for industrial deployment, where minimizing variability is as important as achieving high accuracy. Table 7 highlights the best-performing fold for each model based on macro-averaged F1-score. Both WeldVGG and MobileNetV3 achieved near-perfect classification (F1 = 0.9996), confirming their robustness and reliability. Among the traditional baselines, Random Forest was the strongest, but the gap to the CNN models remained substantial.

**Figure 10:**
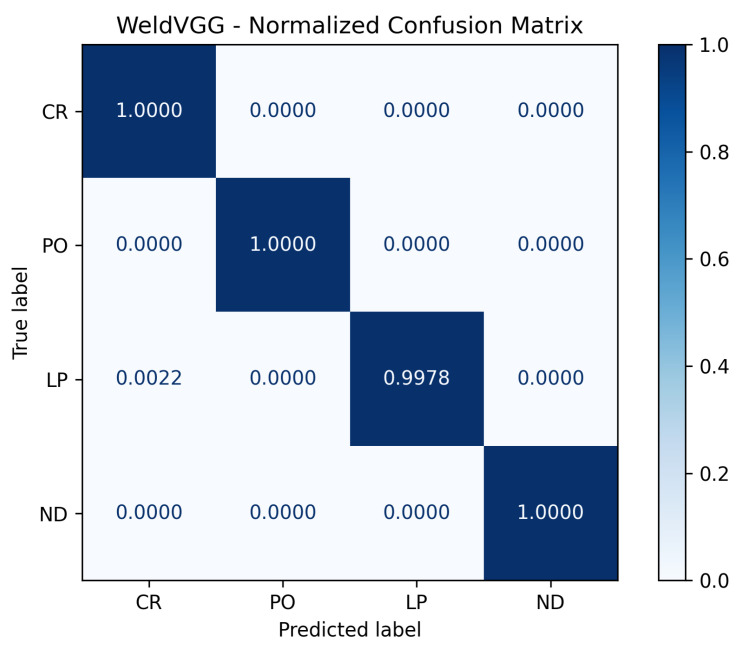
Normalized confusion matrix on a scale from 0 to 1 for the best performing WeldVGG model tested on the RIAWELC dataset.

**Figure 11:**
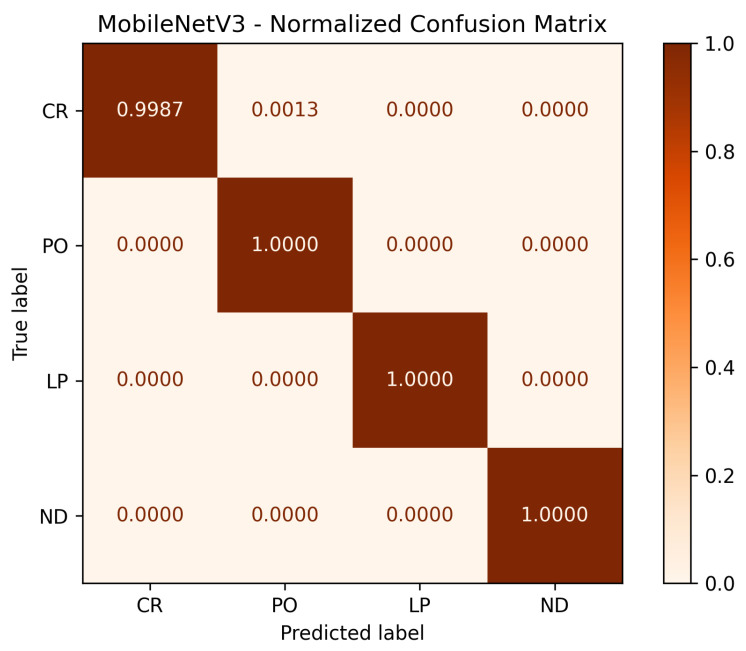
Normalized confusion matrix for the best performing MobileNetV3 model tested on the RIAWELC dataset.

To further analyze per-class performance, Figure 10 and Figure 11 display the normalized confusion matrices corresponding to the best-performing fold for the WeldVGG and MobileNetV3 models, respectively. Both CNNs achieved flawless recognition of the no defect (ND) and porosity (PO) classes, underscoring their ability to reliably distinguish these categories. WeldVGG showed a single instance of confusion between lack of penetration (LP) and cracking (CR), while MobileNetV3 produced one minor misclassification between CR and PO. The WeldVGG’s errors to correctly classify those defects can be explained due to the model confusing lack of penetration with cracks on the radiography. Overall, both models demonstrated near-perfect per-class discrimination, with WeldVGG exhibiting slightly higher sensitivity to LP and MobileNetV3 displaying marginally more consistent separation between CR and PO. These results confirm the robustness of both architectures in accurately detecting weld defects at the class level.

Although both CNNs achieved near-perfect performance on the RIAWELC dataset, these results may not directly translate to unconstrained industrial environments. RIAWELC, while realistic and expert-annotated, is composed of cropped regions of interest with clearly delineated defect patterns. Such conditions inherently reduce background noise and imaging variability, making the classification task more tractable than real-world inspections. To address these limitations, the following sections first examine computational complexity and statistical robustness, before turning, in Section 4.9, to a dedicated cross-dataset evaluation on GDXray to assess generalization.

### 4.6. Computational Complexity Analysis

Computational efficiency is a critical factor for industrial deployment of deep learning models, where resource constraints, real-time requirements, and cost considerations often determine practical feasibility [17]. To comprehensively evaluate the scalability of the proposed WeldVGG architecture, we conduct both empirical measurements and theoretical complexity analysis, comparing against the lightweight MobileNetV3 baseline across multiple performance dimensions.

#### 4.6.1. Empirical Analysis

We measured the computational complexity of WeldVGG against MobileNetV3 under a standardized protocol (input 224×224, batch size =1). We report model size as number of parameters and on-disk size (MB); runtime via single-image latency (ms) and throughput (images/s); and memory footprint via peak GPU memory (VRAM) during inference and training, alongside training efficiency (mean images/s, mean time per epoch, and total training time).

Results show that MobileNetV3 exhibits superior computational efficiency across all reported metrics, as expected for a proven lightweight architecture intended for mobile and embedded systems. As Table 8 shows, WeldVGG contains approximately ∼37× more parameters than MobileNetV3 (56.20 M vs. 1.52 M), reflecting higher representational capacity at a pronounced efficiency cost. Regarding model size, WeldVGG is ∼36× larger on disk (214.39 MB vs. 5.93 MB). By contrast, runtime gaps are modest: single-image latency for WeldVGG is only ∼8% slower (5.77 ms vs. 5.35 ms), and throughput is ∼7% lower (173 vs. 187 img/s). The principal penalty lies in memory usage, where peak inference VRAM rises from 54 MB to 916 MB (∼17×), and overall training cost increases, with total training time about ∼3× longer (8695 s vs. 2924 s). These results show that, as expected, MobileNetV3 represents a better alternative to WeldVGG in real industrial settings where these computational costs translate to higher scalability and adaptability requirements.

Training-time measurements reinforce the same trend in computational efficiency. As summarized in Table 9, WeldVGG sustains only ∼33% of MobileNetV3’s mean training throughput (68.30 vs. 205.25 img/s), with a similar gap at the end of training (∼32%; 68.81 vs. 213.15 img/s). This reduced throughput is accompanied by a substantially higher memory footprint: peak training VRAM increases from 542 MB to 3887 MB (∼7.2×). Consequently, the mean time per epoch is nearly ∼3× longer for WeldVGG (289.83 s vs. 97.48 s). Collectively, these results indicate that MobileNetV3 offers markedly better training scalability and resource efficiency, attributes that are critical for rapid iteration cycles and cost-effective deployment in industrial pipelines, whereas WeldVGG demands disproportionately greater compute and memory resources during optimization.

#### 4.6.2. Theoretical Complexity Analysis

To provide mathematical foundation for the empirical observations, we analyze the computational complexity of both architectures from first principles. The computational cost of a convolutional layer *ℓ* with input feature map $H_{\ell }\times W_{\ell }\times C_{\ell ,in}$, kernel size $K_{\ell }\times K_{\ell }$, and $C_{\ell ,out}$ output channels is as follows:

$$C_{\mathrm{conv}}\left(\ell \right)=H_{\ell }\cdot W_{\ell }\cdot K_{\ell }^{2}\cdot C_{\ell ,in}\cdot C_{\ell ,out}.$$

Fully connected layers require the following:

$$C_{\mathrm{fc}}=N_{\mathrm{in}}\cdot N_{\mathrm{out}}.$$

For WeldVGG (input 224×224×3), the per-block FLOPs are as follows:

(2) $$\begin{array}{cc} \mathrm{Block}\;1: & \;224^{2}\cdot 3^{2}\cdot 3\cdot 64+224^{2}\cdot 3^{2}\cdot 64\cdot 64\approx 1.94\times 10^{9} \end{array}$$

(3) $$\begin{array}{cc} \mathrm{Block}\;2: & \;112^{2}\cdot 3^{2}\cdot 64\cdot 128+112^{2}\cdot 3^{2}\cdot 128\cdot 128\approx 1.18\times 10^{9} \end{array}$$

(4) $$\begin{array}{cc} \mathrm{Block}\;3: & \;56^{2}\cdot 3^{2}\cdot 128\cdot 256+56^{2}\cdot 3^{2}\cdot 256\cdot 256\approx 0.59\times 10^{9} \end{array}$$

(5) $$\begin{array}{cc} \mathrm{Block}\;4: & \;28^{2}\cdot 3^{2}\cdot 256\cdot 512+28^{2}\cdot 3^{2}\cdot 512\cdot 512\approx 0.29\times 10^{9} \end{array}$$

The fully connected layers add the following:

(6) $$\begin{array}{cc} \mathrm{FC}1: & \;100,352\times 512=5.14\times 10^{7} \end{array}$$

(7) $$\begin{array}{cc} \mathrm{FC}2: & \;512\times 256=1.31\times 10^{5} \end{array}$$

(8) $$\begin{array}{cc} \mathrm{FC}3: & \;256\times 4=1.02\times 10^{3} \end{array}$$

Thus, a single forward pass of WeldVGG requires the following:

$$C_{\mathrm{total}}\approx 4.0\times 10^{9}\;\mathrm{FLOPs}\;(∼4\;\mathrm{GFLOPs}).$$

This theoretical estimate aligns with established complexity benchmarks for VGG-style architectures at 224 × 224 resolution, validating our architectural analysis.

For memory complexity, model parameters occupy $56.2\times 10^{6}\times 4$ bytes ≈225 MB (32-bit precision). During training with Adam optimizer, two additional momentum tensors approximately triple this to ∼675 MB. Adding intermediate activations (e.g., the largest feature map: $224^{2}\times 64\times 4\approx 12.8$ MB) and PyTorch framework overhead explains the measured peak of 916 MB inference VRAM and 3887 MB training VRAM.

In contrast, MobileNetV3 employs depthwise separable convolutions, whose complexity is as follows:

$$C_{\mathrm{sep}}=H\cdot W\cdot \left(K^{2}\cdot C_{\mathrm{in}}+C_{\mathrm{in}}\cdot C_{\mathrm{out}}\right),$$

which reduces computational cost by approximately an order of magnitude compared to standard convolutions: $C_{\mathrm{conv}}=H\cdot W\cdot K^{2}\cdot C_{\mathrm{in}}\cdot C_{\mathrm{out}}$. This theoretical efficiency gap directly explains the empirical measurements, where WeldVGG requires ∼17× more inference memory and ∼3× longer training time, confirming the practical implications of architectural design choices for industrial deployment scenarios.

### 4.7. Statistical Validation of CNN Performance

To assess whether the observed performance differences between classifiers were statistically meaningful, pairwise comparisons were conducted using the Wilcoxon signed-rank test [37] over four macro-averaged metrics: Accuracy, F1-Score, Precision, and Recall. Each comparison involved a one-sided test, evaluating whether each convolutional architecture (WeldVGG or MobileNetV3) significantly outperformed the baseline models across 10 independent cross-validation folds.

Table 10 summarizes the directional trend of performance differences, their consistency across all metrics, and whether statistical significance was achieved at the α=0.05 level. Complementary *p*-values for each metric and comparison are provided in Table 11 for transparency and detailed inspection.

These results confirm that both CNN architectures (WeldVGG and MobileNetV3) deliver statistically significant improvements over shallow baselines consistently across all evaluation metrics. However, the pairwise comparison between the two deep models did not yield statistical significance (p>0.05 in both directions), despite MobileNetV3 showing slightly higher average scores. This suggests that, while the deeper WeldVGG maintains state-of-the-art performance, a compact architecture such as MobileNetV3 can achieve comparable results with reduced computational cost, offering a competitive lightweight alternative for industrial deployment.

### 4.8. Comparison with Related Works on RIAWELC

Beyond comparisons with shallow baselines, it is also essential to situate these findings within the context of recent state-of-the-art approaches applied to the same RIAWELC dataset. Table 12 summarizes representative methods, their architectural characteristics, parameter counts, and reported test performance.

This comparison highlights that, while several CNN-based approaches have achieved high accuracy on RIAWELC, improvements to these architectures can still be achieved, as proven by the results of this work’s MobileNetV3 and WeldVGG. Both of these approaches managed to reach over 99.9% accuracy on the testing phase with vastly different parameters size. Through these results, it is possible to conclude that further refinements to CNN-based approaches remain relevant and, particularly, that WeldVGG stands as a solid alternative to state-of-the-art models.

### 4.9. Generalization to External Datasets (GDXray)

Although the proposed architecture achieves near-perfect performance on RIAWELC, such results may not directly translate to unconstrained industrial environments. RIAWELC consists of cropped regions of interest with clearly delineated defect patterns, which reduces background noise and standardizes image conditions. In real-world inspections, however, radiographs often vary substantially in quality, acquisition parameters, and defect appearance. It is therefore essential to evaluate whether the proposed models generalize beyond the dataset on which they were trained.

To this end, we conducted cross-dataset experiments using the weld subset of the publicly available GDXray database [34]. Two scenarios were considered:

- Zero-shot evaluation: Models trained exclusively on RIAWELC were directly tested on GDXray without fine-tuning. This setting simulates deployment to novel inspection environments without access to retraining data.
- Few-shot adaptation: A small number of labeled GDXray images (5 and 10 per class, respectively) were used for fine-tuning, after which the models were evaluated on the remaining GDXray samples. This scenario reflects practical situations in which limited annotation budgets are available for adapting to new factories.

Table 13 summarizes the mean accuracy, macro-F1, macro-Precision and macro-Recall performance across the 10 trained models per scenario.

The results confirm the anticipated domain shift: when transferred directly, both CNNs exhibited a substantial performance drop relative to their near-perfect RIAWELC scores, with macro-F1 decreasing to approximately 64%. This gap highlights the distributional differences between curated datasets and heterogeneous industrial radiographs, and underscores that the near-perfect scores on RIAWELC are not simply a result of overfitting but of dataset homogeneity. Nevertheless, the zero-shot evaluation demonstrates that both models retain transferable representations, avoiding collapse to random performance.

In the few-shot setting, the benefits of limited supervision are evident. With as few as five labeled samples per class, WeldVGG improved by more than 10 percentage points in both accuracy and F1, while MobileNetV3 achieved more modest gains. When ten samples per class were available, WeldVGG reached 83.5% accuracy and 83.4 macro-F1, substantially narrowing the gap to in-domain performance. These results indicate that deeper architectures are better able to exploit limited supervision for rapid domain adaptation, whereas lightweight models such as MobileNetV3 plateau at lower levels of transfer performance.

Overall, these findings reinforce the robustness of the proposed VGG-CNN: despite severe domain shift, it adapts effectively with minimal annotation effort. This suggests that, in realistic factory deployments, the cost of re-annotation can be kept low without sacrificing classification reliability. At the same time, the persistent zero-shot performance drop underscores the need for future research on domain generalization and synthetic augmentation strategies [39], to ensure consistent weld defect detection under highly variable field conditions.

### 4.10. Interpretability Analysis via Grad-CAM++

**Figure 12:**
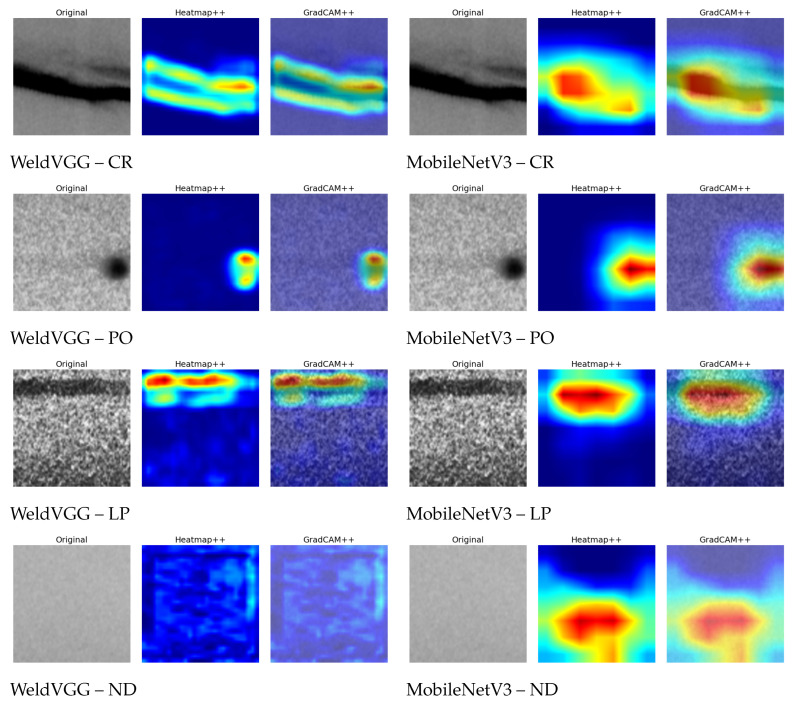
Comparison of Grad-CAM++ visualizations between the WeldVGG and MobileNetV3 models for each class. Each image includes the original weld radiograph, the Grad-CAM++ heatmap, and the overlay. The heatmaps represent the focus of the model on that part of the radiography.

Figure 12 presents a qualitative comparison of Grad-CAM++ visualizations for representative samples from each class, as generated by the WeldVGG and MobileNetV3 models. Visually, both models tend to focus on similar discriminative regions within each defect type, indicating that they have learned to attend to class-relevant patterns in the radiographs. However, the MobileNetV3 consistently produces more intense heatmaps, especially for the cracking (CR), porosity (PO), and lack of penetration (LP) classes. This is reflected in the prevalence of red regions in its saliency maps, suggesting higher attribution scores in those areas. In contrast, the WeldVGG exhibits less sharply defined heatmaps, with comparatively weaker activations and broader, more diffuse attention.

An interesting distinction is observed for the ND class, where the WeldVGG’s Grad-CAM++ map is nearly empty, indicating the absence of dominant localized features, which is consistent with the homogeneous and low-texture nature of ND samples. The MobileNetV3, however, displays residual activations and mild noise across the image, suggesting less confident or less focused reasoning in the absence of defect-specific features. This distinction showcases an important difference between both models, highlighting the ability of the proposed WeldVGG to suppress spurious responses in negative samples (ND) and reserve saliency for truly discriminative cues. In other words, the WeldVGG has a better understanding of the ND class as “absence of defect”.

To complement the qualitative Grad-CAM++ visualizations, we computed the ROADCombined metric as a scalar measure of interpretability fidelity. This metric quantifies how strongly the regions highlighted by saliency maps influence the model’s decisions, with higher values indicating greater alignment between attribution and prediction. Table 14 reports both class-wise and overall ROAD scores across the 2443-image holdout test set, providing a detailed view of explanation quality under in-domain conditions.

The results highlight important differences in attribution behavior between the two models. MobileNetV3 attains higher mean ROAD scores overall (0.272 vs. 0.204), particularly in the defect classes CR (0.330), PO (0.387), and LP (0.381). These values suggest that its Grad-CAM++ maps concentrate more strongly on regions that influence predictions. However, this comes at the expense of stability: MobileNetV3 also exhibits a larger standard deviation (0.198 vs. 0.192), reflecting greater variability across samples and the presence of negative scores in its distribution.

In contrast, WeldVGG produces lower mean ROAD values but with greater consistency across images. This pattern indicates more conservative attribution, where the highlighted regions may be smaller but are more reliably tied to model decisions. This conservatism is especially evident in the ND class, where WeldVGG produces nearly empty saliency maps and a near-zero ROAD score (−0.011±0.054). While this initially appears unfavorable, it is in fact desirable for defect-free samples, since no specific region should dominate the explanation. As such, negative values arise when removing the highlighted regions does not harm (or even improves) the prediction compared to random, implying that the attribution has captured non-causal or counter-evidential content.

Taken together, these findings suggest a trade-off between attribution strength and consistency. MobileNetV3 yields higher ROAD values in defect classes, but may overemphasize or even spuriously activate irrelevant regions, inflating its scores without improving interpretability. WeldVGG, on the other hand, favors steadier and more calibrated explanations that better reflect the presence or absence of discriminative features.

To assess whether these attribution patterns persist beyond the training domain, we further computed ROADCombined scores on the weld subset of the GDXray database. As described in Section 4.9, models were evaluated under zero-shot transfer and with limited few-shot adaptation. The resulting scores are presented in Table 15. This cross-dataset analysis provides a more stringent test of interpretability, since models are confronted with images acquired under different conditions and annotation protocols.

The GDXray evaluation in Table 15 reveals that both models achieve ROAD scores in a similar range to the in-domain setting, but with greater variability and less stable trends. In the zero-shot case, MobileNetV3 again obtains slightly higher overall scores (0.239±0.205) compared to WeldVGG (0.228±0.216), largely due to stronger attribution in the defect classes CR and LP. However, this advantage diminishes under few-shot adaptation. In fact, WeldVGG surpasses MobileNetV3 in the 10-shot setting (0.235 vs. 0.232 overall), indicating that its attribution patterns adapt more effectively to limited supervision.

Across both models, the ND class remains close to zero, with values ranging from 0.019 to 0.087. As in the RIAWELC experiments, this behavior is consistent with the expectation that defect-free images should not trigger concentrated attention. At the same time, the small positive or negative fluctuations highlight the sensitivity of ROADCombined to noise when saliency maps contain little or no structure.

Taken together, these cross-dataset results suggest that while MobileNetV3 tends to achieve higher ROAD scores in defect classes, WeldVGG provides more stable and calibrated explanations when limited adaptation data are available. More importantly, the inconsistent trends across zero-shot and few-shot conditions reinforce the limitations of ROADCombined as a standalone measure of interpretability: higher values do not necessarily correspond to more faithful or trustworthy explanations, particularly when evaluating transferability across domains.

### 4.11. Ablation Studies

To further examine the contribution of key architectural and training choices, we conducted a set of ablation experiments on the RIAWELC dataset. For consistency with the main evaluation protocol, results are reported on Fold 4, which was identified as the best-performing fold for WeldVGG as shown in Table 7. Each ablation modifies a single component of the baseline configuration (four convolutional blocks, two fully connected layers of 512 and 256 units, dropout p=0.5, RGB input at 224×224 with normalization), while all other settings are kept constant. The baseline corresponds to the original WeldVGG architecture described in Section 3.4.

The evaluated variants were as follows:

- Shallow-CNN, reducing the depth to three convolutional blocks.
- No-Dropout, removing dropout layers in the classifier.
- Narrow-FC, decreasing the dense layers to 256 and 128 units.
- Low-res 192, reducing input size from 224×224 to 192×192.
- Grayscale-224, switching from RGB triplication to single-channel input.
- No-Norm, omitting input normalization.

The results shown in Table 16 highlight that both model depth and dropout regularization have measurable impact. Reducing to three convolutional blocks or removing dropout led to small but consistent decreases in macro-F1 (−0.35% and −0.14%, respectively). In contrast, narrowing the fully connected layers or lowering input resolution had negligible effect, indicating redundancy in dense layer capacity and robustness to modest input scaling. Switching to grayscale yielded identical results to the baseline, which is expected given that RIAWELC images are inherently grayscale and the RGB triplication in the baseline adds no new information. Finally, removing normalization also produced virtually no change, suggesting that the dataset’s pixel distribution is already well-conditioned. Overall, these findings confirm the importance of WeldVGG’s depth and regularization, while demonstrating resilience to input modality and resolution variations.

## 5. Conclusions and Future Work

This work presented WeldVGG, a VGG-inspired convolutional model for radiographic weld-defect classification, paired with an interpretability pipeline that combines Grad-CAM++ saliency maps and ROAD scores. On the RIAWELC benchmark, both WeldVGG and the lightweight MobileNetV3 achieved near-perfect performance (macro-F1≈0.999), and Wilcoxon tests confirmed statistically significant gains over shallow baselines. Although MobileNetV3 attained slightly higher averages, differences with WeldVGG were not statistically significant, indicating that compact CNNs can match deeper custom models under controlled conditions.

To assess robustness, we probed cross-dataset generalization on GDXray (series W0003). Zero-shot transfer produced a marked drop (∼64% macro-F1), revealing domain shift between curated tiles and heterogeneous radiographs. With only 10 labeled samples per class for adaptation, WeldVGG recovered to 83.5% accuracy and 83.4 macro-F1, showing that deeper models can leverage limited supervision for practical deployment in new settings. Interpretability results showed complementary behaviors: MobileNetV3 tended to yield stronger but less stable ROAD scores, whereas WeldVGG provided steadier, more calibrated attributions—notably near-empty maps on “no defect” samples—highlighting the value of using quantitative (ROAD) and qualitative (Grad-CAM++) evidence together.

Building on these findings, we outline four avenues to strengthen deployability:

- Localization/segmentation. Complement classification with defect localization (e.g., U-Net/DeepLab or weakly supervised CAM-guided masks) to provide spatial evidence and enable region-level acceptance criteria.
- Open-set and “no-weld” handling. Integrate a front-end weld-presence detector (simple seam detection or a binary segmenter) and open-set/OOD mechanisms (confidence thresholds with ODIN or energy-based scores) to safely reject non-weld or out-of-distribution inputs.
- Generalization and data economy. Combine domain adaptation and physics-aware synthetic augmentation [39] to mitigate domain shift and reduce labeling costs, and broaden validation on additional public/industrial sets.
- Efficiency and usability. Report FLOPs and pursue pruning/quantization or distillation from WeldVGG to MobileNet-class backbones for edge deployment; investigate technician-centric visualization templates aligned with AWS/ISO audit practices.

Together, these directions will help translate the proposed model and interpretability pipeline from a high-accuracy prototype into a trustworthy and auditable industrial tool.

## Figures and Tables

**Table 1:** Layer-by-layer summary of the proposed WeldVGG architecture.

Layer	Type	Kernel/Units	Stride/Pool	Output Size
Input	–	–	–	224 × 224 × 3
Conv Block 1	Conv2d + ReLU × 2	64 × 3 × 3	Stride 1	224 × 224 × 64
	MaxPool2d	–	2 × 2	112 × 112 × 64
Conv Block 2	Conv2d + ReLU × 2	128 × 3 × 3	Stride 1	112 × 112 × 128
	MaxPool2d	–	2 × 2	56 × 56 × 128
Conv Block 3	Conv2d + ReLU × 2	256 × 3 × 3	Stride 1	56 × 56 × 256
	MaxPool2d	–	2 × 2	28 × 28 × 256
Conv Block 4	Conv2d + ReLU × 2	512 × 3 × 3	Stride 1	28 × 28 × 512
	MaxPool2d	–	2 × 2	14 × 14 × 512
Flatten	–	–	–	100,352
FC1	Linear + ReLU	512	–	512
	Dropout	–	*p* = 0.5	512
FC2	Linear + ReLU	256	–	256
	Dropout	–	*p* = 0.5	256
FC3 (Output)	Linear	4	–	4

**Table 2:** Alignment of RIAWELC defect classes with AWS D1.1, ISO 17636-1, and ISO 10675-1 standards.

Class	AWS D1.1	ISO 17636-1	ISO 10675-1
Cracks (CR)	Always rejectable	Linear indications, not acceptable	Not permitted at any level
Porosity (PO)	Limited acceptance by size/density	Cavities/voids detectable	Allowed within limits (Level 1–3)
Lack of Penetration (LP)	Rejectable defect	Incomplete fusion/penetration	Not permitted
No Defect (ND)	Acceptable	No relevant indications	Acceptable

**Table 3:** Class distribution and dataset splits. The dataset was initially stratified by class into 65% training, 25% validation, and 10% testing subsets.

Defect Type	Total	Train	Validation	Test
Cracking (CR)	7635	4962	1908	765
Porosity (PO)	6320	4108	1580	632
Lack of Penetration (LP)	4452	2893	1113	446
No Defect (ND)	6000	3900	1500	600
Total	24,407	15,863	6101	2443

**Table 4:** Class distribution in GDXray dataset.

Defect Type	Total
Cracking (CR)	30
Porosity (PO)	30
Lack of Penetration (LP)	30
No Defect (ND)	30
Total	120

**Table 5:** Summary of experimental settings.

Category	Parameter/Method	Value / Details	Architecture Type
Data Preprocessing	Image Resizing	150×150 pixels	Traditional
	Standardization	StandardScaler	Traditional
	Dimensionality Reduction	PCA (100 components)	Traditional
Model Training	Evaluation Protocol	10-fold stratified cross-validation	All
	Optimizer	Adam	CNN
	Scheduler	None	N/A
	Early stopping	None	N/A
	Learning Rate	$1\times 10^{-4}$	CNN
	Loss Function	Cross-entropy	CNN
	Epochs	30 per fold	CNN
	Batch Size	32	CNN
Hyperparameter Tuning	Method	GridSearchCV	Traditional
	CV for Tuning	5-fold cross-validation	Traditional
	Optimization Criterion	Best F1-score	All

**Table 6:** Summary of macro-averaged metrics across models. Each value is the mean ± standard deviation over 10 runs.

Model	F1 (Mean)	F1 (Std)	Precision (Mean)	Precision (Std)	Recall (Mean)	Recall (Std)
MobileNetV3	0.9989	0.0005	0.9989	0.0005	0.9989	0.0005
WeldVGG	0.9987	0.0005	0.9988	0.0006	0.9986	0.0005
Random Forest	0.9893	0.0013	0.9900	0.0015	0.9887	0.0012
KNN	0.9801	0.0030	0.9799	0.0031	0.9806	0.0028
SVC	0.9638	0.0016	0.9647	0.0017	0.9634	0.0015
Decision Tree	0.8030	0.0079	0.8030	0.0073	0.8051	0.0090

**Table 7:** Best run per model based on F1 score, with corresponding macro-averaged metrics.

Model	Fold	Accuracy	F1	Precision	Recall
MobileNetV3	3	0.9996	0.9996	0.9996	0.9997
WeldVGG	4	0.9996	0.9996	0.9997	0.9994
Random Forest	8	0.9906	0.9910	0.9920	0.9901
KNN	5	0.9828	0.9838	0.9836	0.9842
SVC	5	0.9673	0.9670	0.9680	0.9665
Decision Tree	7	0.8174	0.8165	0.8140	0.8226

**Table 8:** Inference and model size comparison at 224 × 224 (batch size = 1).

Metric	MobileNetV3	WeldVGG	Ratio
Parameters (M)	1.522	56.198	36.93
Model size (MB)	5.93	214.39	36.15
Single-image latency (ms)	5.347	5.769	1.08
Throughput (img/s)	187.04	173.33	0.93
Peak inference VRAM (MB)	54	916	16.96
Total training time (s)	2924.48	8694.80	2.97

**Table 9:** Training throughput and memory profile (mean over epochs).

Metric	MobileNetV3	WeldVGG
Training throughput (img/s, mean)	205.25	68.30
Training throughput (img/s, last epoch)	213.15	68.81
Peak training VRAM (MB)	542	3887
Train time per epoch (s, mean)	97.48	289.83

**Table 10:** Summary of pairwise comparisons with Wilcoxon signed-rank test across performance metrics.

CNN Model	Model Compared	Trend	Consistent Across Metrics	Statistically Significant
WeldVGG	Decision Tree	WeldVGG > Decision Tree	Yes	Yes (p<0.05)
WeldVGG	Random Forest	WeldVGG > Random Forest	Yes	Yes (p<0.05)
WeldVGG	KNN	WeldVGG > KNN	Yes	Yes (p<0.05)
WeldVGG	SVC	WeldVGG > SVC	Yes	Yes (p<0.05)
WeldVGG	MobileNetV3	WeldVGG < MobileNetV3	Yes	No (p≈0.768)
MobileNetV3	Decision Tree	MobileNetV3 > Decision Tree	Yes	Yes (p<0.05)
MobileNetV3	Random Forest	MobileNetV3 > Random Forest	Yes	Yes (p<0.05)
MobileNetV3	KNN	MobileNetV3 > KNN	Yes	Yes (p<0.05)
MobileNetV3	SVC	MobileNetV3 > SVC	Yes	Yes (p<0.05)
MobileNetV3	WeldVGG	MobileNetV3 > WeldVGG	Yes	No (p≈0.258)

**Table 11:** Wilcoxon signed-rank test *p*-values for accuracy, F1, precision, and recall across model pairs.

Model Compared	WeldVGG > Other				MobileNetV3 > Other			
	Accuracy	F1	Precision	Recall	Accuracy	F1	Precision	Recall
Decision Tree	0.001	0.001	0.001	0.001	0.001	0.001	0.001	0.001
Random Forest	0.001	0.001	0.001	0.001	0.001	0.001	0.001	0.001
KNN	0.001	0.001	0.001	0.001	0.001	0.001	0.001	0.001
SVC	0.001	0.001	0.001	0.001	0.001	0.001	0.001	0.001
MobileNetV3	0.737	0.812	0.688	0.833	—	—	—	—
WeldVGG	—	—	—	—	0.290	0.216	0.348	0.180

**Table 12:** Comparison of weld defect classification performance on the RIAWELC dataset with previously published works. Reported results are those cited in the corresponding papers.

Reference	Model	Dataset	Params (M)	Test Accuracy
[30]	CNN (RIAWELC release)	RIAWELC	∼0.69	93.33
[31]	SqueezeNet V1.1	RIAWELC	∼0.72	99.8
[31]	WelDeNet	RIAWELC	∼0.39	99.5
[4]	ResNet50	RIAWELC	∼23	98.24
[38]	VGG16	Custom	Not specified	97.6
This work	MobileNetV3	RIAWELC	∼1.5	**99.9**
This work	WeldVGG	RIAWELC	∼56	**99.9**

**Table 13:** Detailed GDXray cross-dataset results (mean ± std over 10 runs). Macro-averaged metrics are emphasized as they account for class imbalance.

Model	Scenario	Accuracy	F1 Macro	Precision Macro	Recall Macro
MobileNetV3	Zero-shot	63.8 ± 3.5	64.0 ± 3.2	66.2 ± 3.3	63.8 ± 3.5
	Few-shot (5/cl.)	67.3 ± 4.5	67.7 ± 4.7	71.2 ± 4.1	67.3 ± 4.5
	Few-shot (10/cl.)	76.6 ± 4.2	76.5 ± 4.3	81.0 ± 2.1	76.6 ± 4.2
WeldVGG	Zero-shot	64.2 ± 4.3	63.0 ± 4.7	65.6 ± 4.7	64.2 ± 4.3
	Few-shot (5/cl.)	73.7 ± 3.4	73.9 ± 3.2	76.4 ± 3.6	73.7 ± 3.4
	Few-shot (10/cl.)	83.5 ± 3.1	83.4 ± 3.2	83.9 ± 3.2	83.5 ± 3.1

**Table 14:** ROADCombined scores across all test images for the WeldVGG and MobileNetV3 models. Higher values indicate stronger faithfulness of Grad-CAM++ explanations to model decisions.

Class	WeldVGG		MobileNetV3	
	Mean ± Std	n	Mean ± Std	n
Cracking (CR)	0.240 ± 0.160	765	0.330 ± 0.141	765
Porosity (PO)	0.340 ± 0.155	632	0.387 ± 0.122	632
Lack of Penetration (LP)	0.240 ± 0.168	446	0.381 ± 0.132	446
No Defect (ND)	−0.011 ± 0.054	600	−0.004 ± 0.040	600
Overall	0.204 ± 0.192	2443	0.272 ± 0.198	2443

**Table 15:** ROADCombined scores on the GDXray weld subset under zero-shot and few-shot adaptation settings. *N* denotes the number of evaluation images after excluding adaptation samples. Values are mean ± standard deviation. Bold values highlight the best performance per class.

Model	Setting	N	CR	PO	LP	ND	Overall
WeldVGG	Zero-shot	120	0.246±0.185	0.330±0.212	0.251±0.231	0.087±0.151	0.228±0.216
	5-shot	100	0.297±0.186	0.386±0.118	0.176±0.250	0.053±0.142	0.228±0.221
	10-shot	80	0.293±0.196	0.343±0.197	0.275±0.144	0.031±0.117	0.235±0.206
MobileNetV3	Zero-shot	120	0.278±0.171	0.337±0.231	0.280±0.156	0.059±0.129	0.239±0.205
	5-shot	100	0.240±0.251	0.363±0.220	0.229±0.175	0.077±0.144	0.227±0.226
	10-shot	80	0.234±0.241	0.387±0.166	0.288±0.179	0.019±0.131	0.232±0.228

**Table 16:** Ablation study on the global test set, conducted on Fold 4. Macro-averaged metrics are reported.

Variant	Accuracy	F1_*macro*_	P_*macro*_	R_*macro*_	F1_*weighted*_	P_*weighted*_	R_*weighted*_
Shallow-CNN	0.9955	0.9952	0.9946	0.9959	0.9955	0.9955	0.9955
No-Dropout	0.9943	0.9942	0.9945	0.9939	0.9943	0.9943	0.9943
Narrow-FC	0.9988	0.9987	0.9987	0.9988	0.9988	0.9988	0.9988
Low-res 192	0.9988	0.9987	0.9986	0.9988	0.9988	0.9988	0.9988
Grayscale-224	0.9996	0.9996	0.9997	0.9994	0.9996	0.9996	0.9996
No-Norm	0.9992	0.9991	0.9993	0.9989	0.9992	0.9992	0.9992
Baseline (WeldVGG)	0.9996	0.9996	0.9997	0.9994	0.9996	0.9996	0.9996

## Data Availability

The datasets analyzed in this study are publicly available. The RIAWELC dataset can be accessed at https://github.com/stefyste/RIAWELC (accessed on 16 September 2025), and the GDXray dataset is available at https://github.com/computervision-xray-testing/GDXray (accessed on 16 September 2025).

## Appendix A. Hyperparameter Tuning Grids

This appendix summarizes the hyperparameter tuning process used for the shallow classifiers. Grid search with 5-fold cross-validation was performed for each model using the parameter ranges listed in Table A1. The best-performing configurations—based on macro-F1 score on the validation folds—are reported in Table A2.

**Table A1:** GridSearchCV parameter ranges for each model.

Model	Parameter	Values
Decision Tree	criterion	gini, entropy
	max depth	None, 10, 20, 30, 50
	min samples split	2, 5, 10
	min samples leaf	1, 2, 4
	splitter	best, random
Random Forest	n estimators	10, 50, 100, 200
	max depth	None, 5, 10, 20, 50
	min samples split	2, 5
	min samples leaf	1, 2, 4
	criterion	gini, entropy
K-Nearest Neighbors	n neighbors	3, 5, 7, 9
	weights	uniform, distance
	*p*	1, 2
	algorithm	auto, ball tree, kd tree, brute
	leaf size	30, 40, 50
	metric	minkowski, euclidean, manhattan
Support Vector Classifier	C	0.1, 1, 10, 100, 200, 500
	gamma	scale, auto, 0.1
	kernel	rbf, linear, poly

**Table A2:** Best parameter configuration selected via GridSearchCV.

Model	Parameter	Value
Decision Tree	criterion	entropy
	max depth	10
	min samples leaf	4
	min samples split	10
	splitter	best
Random Forest	criterion	entropy
	max depth	None
	min samples leaf	1
	min samples split	2
	n estimators	200
K-Nearest Neighbors	algorithm	auto
	leaf size	30
	metric	minkowski
	n neighbors	3
	*p*	2
	weights	distance
Support Vector Classifier	C	500
	gamma	scale
	kernel	rbf
